# A multi-omic single-cell landscape reveals transcription and epigenetic regulatory features of t(8;21) AML

**DOI:** 10.1186/s12967-025-06659-0

**Published:** 2025-07-24

**Authors:** Xue-Ping Li, Yan Gao, Bai-Tian Zhao, Yu-Ting Dai, Jia-Ying Mao, Yang Liang, Lu Jiang

**Affiliations:** 1 Department of Hematologic Oncology, Sun Yat-sen University Cancer Center, Guangzhou, 510060 China; 2 State Key Laboratory of Oncology in South China, Guangdong Provincial Clinical Research Center for Cancer, Sun Yat-sen University Cancer Center, Guangzhou, 510060 China; 3 Department of Medical Oncology, Sun Yat-sen University Cancer Center, Guangzhou, 510060 China; 4 Shanghai Institute of Hematology, State Key Laboratory of Medical Genomics, National Research Center for Translational Medicine at Shanghai, Ruijin Hospital affiliated to Shanghai Jiao Tong University School of Medicine, Shanghai, 200025 China

**Keywords:** scRNA-seq, scATAC-seq, TCR repertoire, T(8;21) AML

## Abstract

**Background:**

Comprehensive analysis of single-cell transcriptome and chromatin accessibility will contribute to interpret the heterogeneity of acute myeloid leukemia (AML). We hypothesize that integrating single-cell transcriptomic and chromatin accessibility landscapes underlying t(8;21) AML will provide valuable insights into its heterogeneous cellular properties and gene regulatory programs.

**Methods:**

Here, we conducted paired single-cell RNA-sequencing (scRNA-seq) and single-cell ATAC sequencing on bone marrow samples from newly diagnosed t(8;21) AML patients and healthy controls. Genetic signatures extracted from scRNA-seq were built and validated across three independent cohorts (German AMLCG1999, GSE106291 and TCGA LAML).

**Results:**

We identified *TCF12*, a core component of AML1-ETO-containing transcription factor complex (AETFC), as the most active transcription factor in blast cells, driving a universally repressed chromatin state. Furthermore, we delineated two functionally distinct T cell subsets, revealing that *EOMES*-mediated transcriptional regulation promotes the expansion of a cytotoxic T cell population (T cells_2; high *GNLY*, *NKG7* and *GZMB* expression), with an increased clonality and a tendency for drug resistance. In addition, we discovered a novel leukemic CMP-like cluster characterized by high *TPSAB1*, *HPGD* and *FCER1A* expression. Leveraging machine learning-based integration of multi-omic profiles, we identified a robust 9-gene prognostic signature, which demonstrated significant predictive value for AML outcomes across three independent cohorts.

**Conclusions:**

This multi-omics study provides unprecedented insights into the transcriptional and epigenetic heterogeneity of t(8;21) AML, providing a potential actionable tool for clinical risk stratification.

**Supplementary Information:**

The online version contains supplementary material available at 10.1186/s12967-025-06659-0.

## Introduction

Acute Myeloid Leukemia (AML) is a heterogeneous and complex hematologic malignancy characterized by a diverse range of cellular subsets, which presents distinct responses to treatment. One of the most common chromosomal abnormalities in AML is the t(8;21) translocation, which produces the RUNX1::RUNX1T1 fusion protein (also known as AML1-ETO). This fusion protein plays a key role in the pathogenesis of AML, and epigenetic dysregulation is a distinctive hallmark of the disease [1].

Previous studies have revealed that the depletion of *RUNX1::RUNX1T1* in leukemic cells would lead to a genome-wide alteration in transcription factor binding, suggesting its critical role of epigenetic reprogramming in t(8;21) AML [2]. Furthermore, *RUNX1::RUNX1T1* resides in leukemic cells and functions through a stable *AML1*-*ETO*-containing transcription factor complex (AETFC), which contains several hematopoietic transcription (co)factors. These AETFC components co-localize genome wide, providing multiple DNA-binding domains for target genes and stabilizing the AETFC complex, thereby contributing to the pathogenesis of t(8;21) AML [3, 4]. Despite AML with t(8;21) translocation being considered with a favorable prognosis, approximately 30–50% of patients experience relapse and drug resistance [5]. The heterogeneity and complexity observed at both the cellular and epigenetic levels of t(8;21) AML present major challenges in curing this disease. We hypothesize that investigating gene expression profiling and chromatin accessibility underlying t(8;21) AML will provide valuable insights into its heterogeneous cellular properties and gene regulatory programs.

Chromatin remodeling events, including alterations of chromatin accessibility and histone modification, have been reported to regulate the gene expression profiling in epigenetic mechanism patterns and are thus closely linked to the disease status of AML. Recent advances in high-throughput technologies, particularly single-cell analyses, have provided great opportunities to interrogate the cellular heterogeneity and global chromatin landscapes in AML. Although several studies have revealed the critical role of aberrant chromatin accessibility in the pathogenesis of hematological malignancies [6, 7], the epigenetic regulatory mechanisms underlying t(8;21) AML at single-cell resolution remain elusive.

To explore the heterogeneous cellular properties and epigenetic cis-regulatory programs of t(8;21) AML, in this study, we performed a comprehensive analysis using single-cell RNA sequencing (scRNA-seq), single-cell assay for transposase-accessible chromatin with sequencing (scATAC-seq), and single-cell T cell receptor sequencing (scTCR-seq) on bone marrow samples from newly diagnosed patients with t(8;21) AML. This multi-omic approach allows for an in-depth examination of gene expression patterns, global chromatin landscapes, and the exact T cell receptor clonotype profiles of T cell subtypes within the tumor microenvironment (TME) of t(8;21) AML. Through systematically comparing the AML samples with those of healthy individuals at the single-cell level, we characterized the substantial inter- and intra-patient heterogeneity, as well as the chromatin accessibility landscape within t(8;21) AML, and revealed a genetic signature that could serve as a novel prognostic indicator across multiple independent AML cohorts.

## Methods

### Human specimens

The bone marrow samples of patients with primary t(8;21) AML were collected in compliance with the Declaration of Helsinki. The detailed clinical information of the samples and treatment regimens is described in Table S1. All patients provided written informed consent, and this study was approved by the Ethics Committee of Ruijin Hospital Affiliated to Shanghai Jiao Tong University School of Medicine (2023-355) and Sun Yat-sen University Cancer Center (G2023-084-01,G2023-180-01).

### Single-cell RNA-seq and V(D)J library preparation and sequencing

Single-cell RNA sequencing and V(D)J libraries for t(8;21) AML samples were prepared using a 10x Single Cell Immune Profiling Solution Kit v2.0 (10x Genomics, 1000263), according to the manufacturer’s instructions. The libraries were subsequently sequenced on a NovaSeq 6000 platform (Illumina).

### Single-cell RNA-seq library processing

Raw sequencing reads of scRNA-seq were processed using Cell Ranger (10x Genomics, default settings, version 5.0.0) and aligned to the human GRCh38 reference genome. Quality control and downstream analysis were performed with the Seurat (v3.0.2) R toolkit [8]. All functions were run with default parameters, unless specified otherwise. We excluded cells with fewer than *200* or more than *6*, *000* genes or with more than 10% mitochondrial RNA genes. The top *2*, *000* highly variable genes were identified. Harmony was employed to adjust sample batch effect. Uniform manifold approximation and projection (UMAP) [9] was performed to visualize the clusters. Cell identities were annotated based on the SingleR software [10] and canonical markers, including *CD34* for progenitor cells, *CD14* for monocytes, *CD3* for T cells, and *CD79A* for B cells.

### Single-cell V(D)J library processing

Raw sequencing reads of scTCR-seq were processed using the Cell Ranger VDJ pipeline (10x Genomics, version 5.0.0) to assemble the TCR sequences. Cells annotated as T cell cluster in the scRNA-seq data and with productive TCR α and β chains were further analyzed. V-J gene combinations in T cells were visualized via Sankey by using the ggplot2 package in R software.

### Functional enrichment and cell-cell communication analysis

Gene Ontology and gene set enrichment analysis (GSEA) of differentially expressed genes were performed by using clusterProfiler R package (v 3.14.0) [11]. Cell-cell communication in primary t(8;21) AML was inferred using the CellChat toolkit [12].

### Single-cell ATAC-seq library preparation

The nuclei were isolated with a Shbio Cell Nuclear Isolation Kit (Shbio, #52009–10). Libraries were constructed by using a Chromium™ Single Cell ATAC GEM, Library & Gel Bead Kit v2.0 (10x Genomics, 1000390) following the manufacturer’s guidelines. Libraries were sequenced with paired-end (2 × 100) by NovaSeq 6000 (Illumina).

### Single-cell ATAC-seq data processing

Cell Ranger-ATAC (version 2.0.0) was used for scATAC-seq data processing. Cells were removed based on the following criteria: a transcriptional start site (TSS) enrichment score lower than 4 and fewer than 3,000 total fragments in peaks, as determined using ArchR [13]. In addition, doublets were filtered out using ArchR software. Harmony was used to remove batch effects cross-samples. Identities of cells from scATAC-seq were annotated from the corresponding scRNA-seq data. A total of 25,491 nuclei (from 3 leukemia samples) and 38,462 nuclei (from 3 leukemia samples and 2 controls) were retained for cross-sample integration analysis. UMAP was used for visualization.

### Integrated analysis of scRNA-seq and paired scATAC-seq

To determine the cluster-specific marker genes, we applied a log2 fold change threshold greater than 1.25 and a false discovery rate (FDR) of less than 0.01 using the Wilcoxon test. For identifying cluster-specific peaks, we conducted pseudo-bulk replicates and applied MACS2 software to display the number of peaks in different regions. Motif deviation enrichment and transcription factor (TF) footprinting were also analyzed. Genomic track profiles of specific regions were visualized with CoveragePlot.

### Construction of the leuk_CMP-like signature from joint integration of scATAC-seq and scRNA-seq data

The genetic markers expressed in the Leuk_CMP-like cluster were calculated through the joint analysis of scATAC-seq and scRNA-seq data. Overexpressed genes were defined as those with an average log2 (fold change) greater than 3.0 and a *p* value below 0.05. A total of 136 genes were identified (Table S2). The German AMLCG1999 dataset was used as the training dataset and LASSO regression analysis was used to construct the final model.

### Data availability

The raw sequencing data reported in this paper have been deposited in the Genome Sequence Archive in National Genomics Data Center (https://ngdc.cncb.ac.cn/gsa-human), under the accession number #HRA007073. Due to the legal restrictions, these data are under controlled access. Requests for access to these data for research purposes can be directed to the corresponding author Dr. Lu Jiang (jl11891@rjh.com.cn).

### Statistical analysis

The statistical analysis of this study was conducted by R software (version 4.2.0).

## Results

### Integrative single-cell analysis discriminated leukemic cells in primary t(8;21) AML

**Fig. 1:**
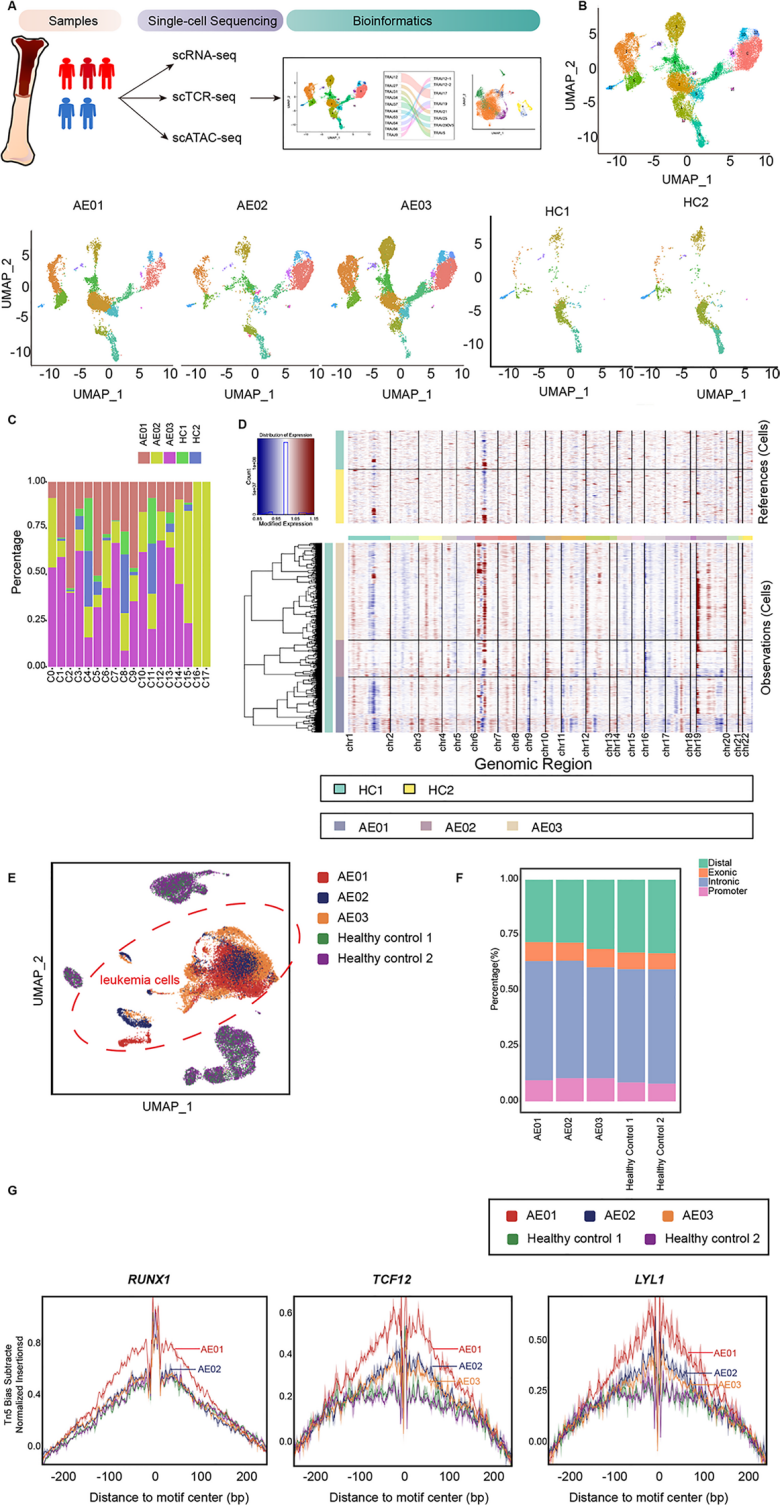
Discrimination of leukemia cells in primary t(8;21) AML samples from single-cell RNA-seq and scATAC-seq. (**A**). Flow chart of the study design. We conducted paired single-cell RNA-sequencing, single-cell TCR repertoire and single-cell ATAC-seq of bone marrow mononuclear cells (BMMCs) from t(8;21) AML patients (*n* = 3) and healthy controls (*n* = 2). (**B**). Uniform manifold approximation and projection (UMAP) plot demonstrated single-cell RNA-seq profile of 32,457 cells from t(8;21) AML (*n* = 3) and healthy controls (*n* = 2). Dots indicates individual cells. (**C**). Bar plot showed the percentage of cells from each sample in each cluster. (**D**). Heatmap displayed inferred copy number variations (CNVs) from t(8;21) AML samples in comparison to healthy controls. (**E**). UMAP demonstrated the integration scATAC-seq analysis of t(8;21) AML (*n* = 3) with healthy controls (*n* = 2). (**F**). Summary of peaks distribution in the t(8;21) AML samples and healthy controls. (**G**). Footprint of leukemia-specific transcription factor *RUNX1*, *TCF12* and *LYL1* in t(8;21)AML and healthy controls

In order to characterize the immune repertoire as well as the chromatin accessibility underlying the bone marrow (BM) heterogeneity of newly diagnosed t(8;21) AML patients, we conducted paired scRNA-seq, scTCR-seq and scATAC-seq analyses with 10x genomics technology. The flow chart of the study was demonstrated in Fig. 1A. The clinical features of the analyzed samples were summarized in Table S1. In brief, we collected a total of 28,000 cells from the bone marrow of AML patients harboring *RUNX1-RUNX1T1* fusion and wild-type *KIT*, with an average of 54,634 mean reads per cell. In addition, we reanalyzed scRNA-seq data as well as scATAC-seq data of normal BM samples from healthy individuals [14, 15] to construct a reference for the hematopoietic lineage as a normal control.

First, to discriminate the leukemic cell identities of the primary t(8;21) AML samples, we adjusted for batch effects and performed an integrated analysis of both AML and normal cells. A total of eighteen cell clusters were identified (Fig. 1B and C and Fig. S1). After combining of automatic annotation and manual verification referring to our previous work [16], we determined the cell identities of the cells clusters. Cluster_4 cells, characterized by high expression of *CD3D* and *LTB*, were annotated as T cells. Cluster_8 cells, exhibiting high expression of *GNLY* and *NKG7*, belonged to NK cells. Cluster_5 cells highly expressing *CD79B* and *DNTT* were annotated as B cells. In addition to elevated expression of *CD79B* and *DNTT*, Cluster_11 cells also presented increased expression of *CD34* and thus were annotated as the progenitors of B cells. Cluster_3 cells and Cluster_13 cells, which highly expressed *S100A8* and *FCN1*, were annotated as monocytes and macrophages, respectively. The remaining clusters, including Cluster_0, Cluster_2, Cluster_6–7, Cluster_9–10, Cluster_12, Cluster_14, Cluster_15-Cluster_17 were composed of leukemic cells. We also identified a small cluster of cells (Cluster_1) that highly expressed the cell-cycle related genes *TYMS*, *UBE2C* and *KIAA0101*, indicating a proliferative state. Interestingly, most of the cycling cells in Cluster_1 derived from leukemia samples, presented a highly proliferative state, whereas very few were presented in the healthy controls. Further exploration of chromosomal copy number variations (CNVs) of t(8;21) AML samples in comparison with healthy controls (Fig. 1D) also revealed recurrent alterations in the majority of leukemic cells in t(8;21)AML samples.

To explore the alterations in chromatin accessibility, we performed scATAC-seq on the same matched samples. Our analysis obtained cis-regulatory data for 28,977 cells (Table S1 and Fig. 1E), with an average of 20,187 unique fragments mapping to the nuclear genome. Approximately 60.2% of the Tn5 insertions were located within aggregate ATAC-seq peaks (Fig. S2A and Fig. S2B), providing a comprehensive view of chromatin accessibility in the t(8;21) AML samples.

We compared the single-cell chromatin landscape of t(8;21) AML samples to that of healthy individuals [15]. Consistent with the findings from the scRNA-seq, leukemia samples exhibited a substantial discrepancy in chromatin accessibility compared to normal controls (Fig. 1E). However, in terms of the distribution of peaks, there was no significant difference between leukemia samples and healthy controls (Fig. 1F).

Next, we focused on the role of the AML1-ETO fusion protein in t(8;21) AML, which is known to act through the AETFC [3, 4]. Specifically, we examined the transcription factors associated with the AETFC, including E proteins *TCF12* (HEB) and *TCF3* (E2A), using the the scATAC-seq data. Compared to healthy controls, t(8;21) AML samples presented increased chromatin accessibility at loci of *TCF12*, *LYL1*, and *RUNX1* (Fig. 1G), indicating the pathogenesis of AML1-ETO in t(8;21) AML.

### Chromatin landscape of cell type-specific gene expression in primary t(8;21) AML

**Fig. 2:**
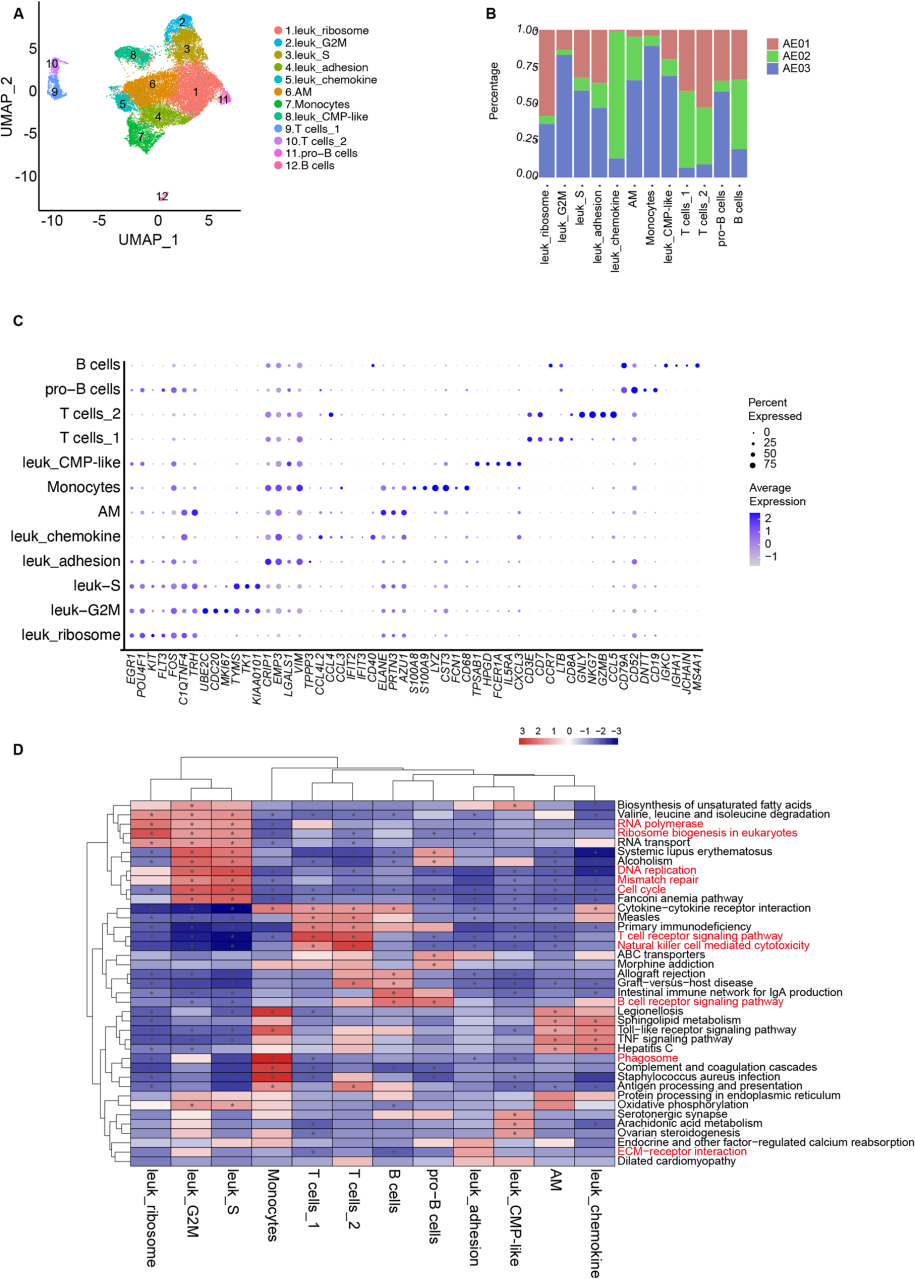
single-cell RNA-seq profiling of the leukemia clusters in primary t(8;21) AML. (**A**). UMAP demonstrated the annotation of each cluster in primary t(8;21) AML samples (*n* = 3). (**B**). Bar plot showed the percentage of each cluster from each sample. (**C**). Dot plot displayed the highly expressed genetic markers of each annotated cell cluster. Dot indicates the percentage of expression in each cluster and color represents the average expression. (**D**). Heatmaps displayed the top specific pathways that were enriched in each cluster

To decipher the characteristics of the leukemic cells in t(8;21) AML, we analyzed the scRNA-seq data from three samples from newly-dignosed patients (*n* = 3) and revealed the major cell clusters (Fig. 2A and B and Fig. S3A). The cell cluster characterized by high expression of *CD79A*, *JCHAIN* and *IGKC*, was characterized as B cells, whereas the cell cluster expressing *CD3D*, *CD5* and *CD7* was identified as T cells (Fig. 2C and S3B). The cells that highly expressed *S100A8*, *S100A9* and *FCN1* were identified as monocytes.

We observed significantly higher expression levels of *RUNX1T1* in leukemic cells than in cells from the tumor microenvironment (TME) (Fig. S3C). Among the leukemic cells, seven distinct cell populations were observed. The cell cluster with high expression levels of *ELANE*, *PRTN3* and *AZU1* was identified as Leuk_abnormal myeloid cells with partial maturation (AM). Cell clusters of leukemic cells at cell cycle of G2M phase (highly expressing *UBE2C* and *CDC20*) and S phase (highly expressing *TYMS* and *TK1*) were annotated as leuk_G2M and leuk_S, respectively (Fig. 2C and Fig. S3D). GSEA analyses revealed significant activation of the cell cycle pathways of these two cell clusters, which was consistent with their respective cell cycle stages (Fig. 2D). Additionally, cell cluster highly expressing *EGR1*, *POU4F1*, *KIT* and *FLT3* was annotated as leuk_ribosome. Pathway analysis of this cluster revealed enrichment of RNA polymerase and ribosome biogenesis in eukaryotes (Fig. 2D). Cell cluster that presented high expression of *CRIP1*, *EMP3* and *LGALS1* was annotated as leuk_adhesion. A small cluster, characterized by high expression of *CCL4* and *CCL3*, was designated leuk_chemokine. Last but not least, a subset of cells with elevated expression of *TPSAB1*, *HPGD* and *FCER1A*, showing a common myeloid progenitor (CMP) signature, was referred to as leuk_CMP-like.

**Fig. 3:**
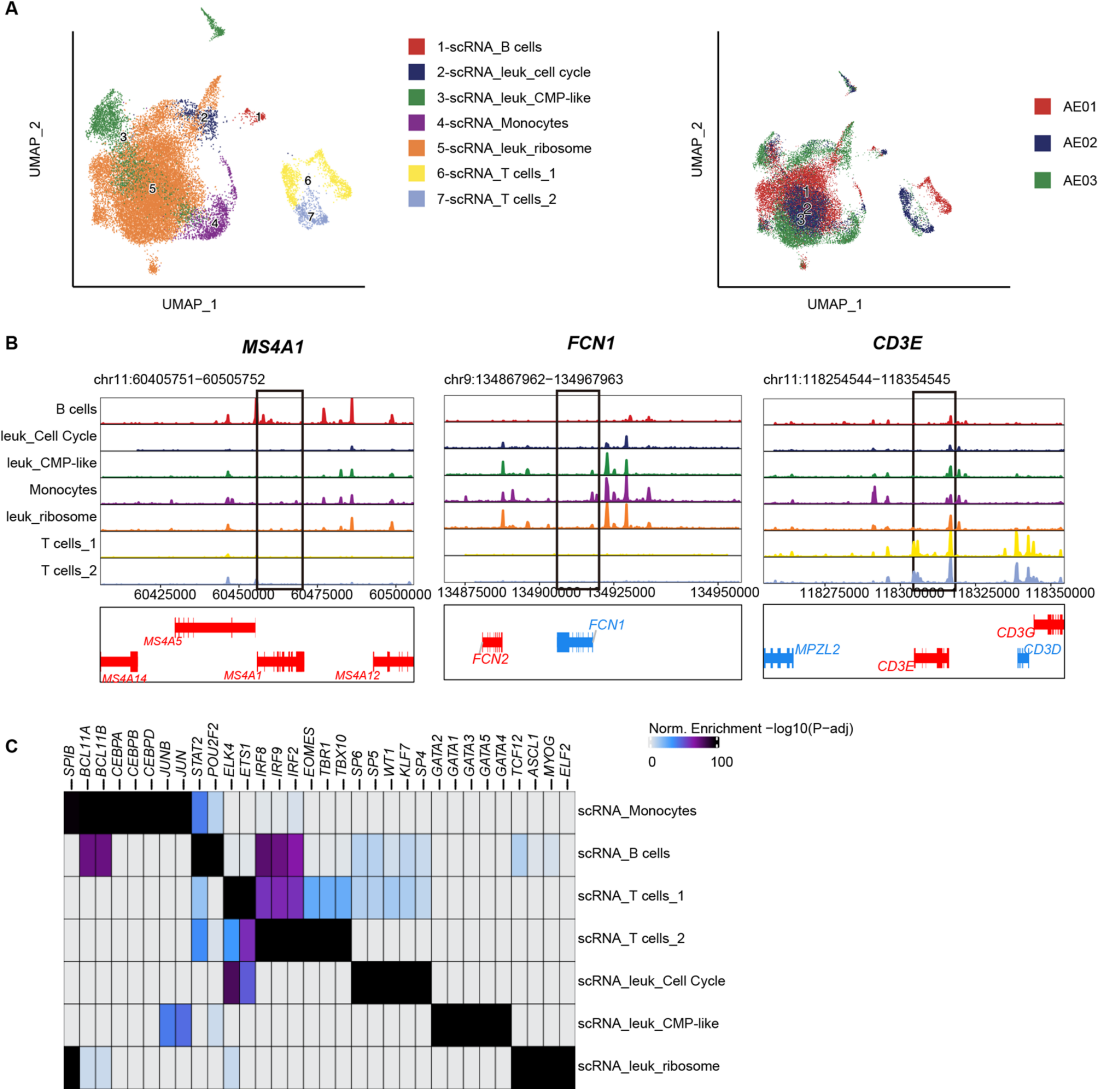
Single-cell chromatin accessibility profiling revealed the cell-cluster specific epigenetic features in t(8;21) AML. (**A**). (Left) UMAP showed the chromatin accessibility of 28,977 single cells from t(8;21) AML (*n* = 3). Dot indicates individual cells. (Right) Projection of leukemia cells in each sample. (**B**). Cell-specific accessible peaks with representative associated genes. Genome tracks showed the chromatin accessibility of *MS4A1* (*CD20*) for B cells, *FCN1* for monocytes and *CD3E* for T cells. (**C**). Heatmaps displayed the differentially expressed transcription factors in identified cell clusters in scATAC-seq

We then investigated the chromatin remodeling across distinct cell clusters and characterized the major cell populations identified by scRNA-seq from bone marrow of primary t(8;21) AML. Cell-specific marker genes derived from scATAC-seq were calculated. We further confirmed the cell identities (Fig. 3). B cells identified through scATAC-seq presented specific enrichment of *MS4A1*, *BLC11A*, and *IRF8* peaks (Fig. S5A), whereas T cells showed notable enrichment of *CD3E*. Monocytes presented specific enrichments of *FCN1*, *CEBPA* and *CEBPB* (Fig. 3B and C and Fig. S5B). These findings further verified the identities of the cell clusters observed in the scRNA-seq data.

### Heterogeneous clonality and chromatin accessibility of T cell subsets

**Fig. 4:**
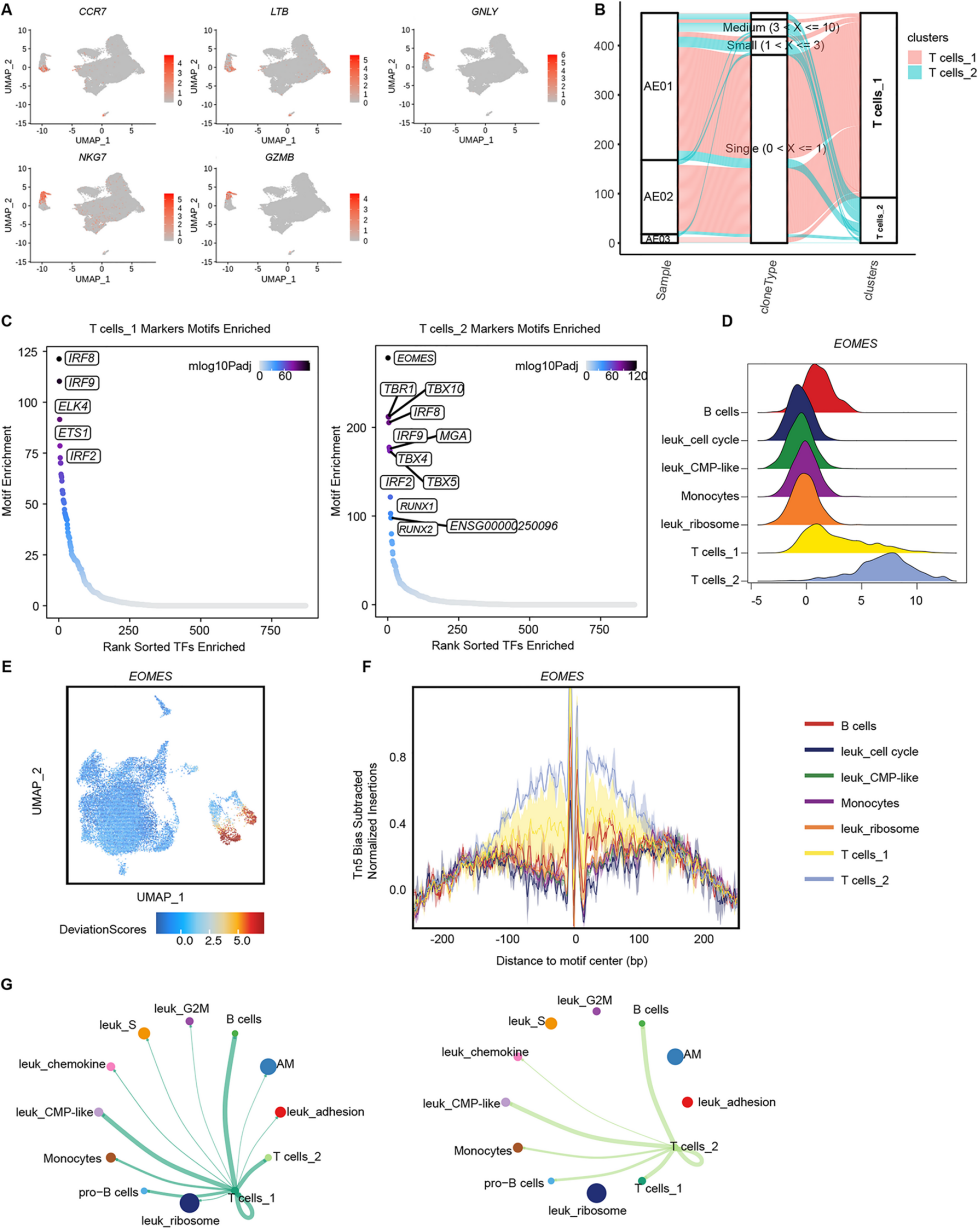
Single-cell TCR repertoire profiling and single-cell ATAC-seq analysis revealed heterogeneous clonality and chromatin accessibility of T cells subsets. (**A**). UMAP displayed the expression of T cells_1 marker genes (*CCR7*, *LTB*) and T cells_2 marker genes (*GNLY*, *NGK7*, *GZMB*) in the scRNA-seq landscape of t(8;21) AML (*n* = 3). (**B**). Clonotypes of the two clusters of T cells in each sample analyzed with scTCR. (**C**). Plots showing the most accessible motifs observed in T cells_1 (*IRF8*, *IRF9*, *ETS1*) and T cells_2 (*EOMES*, *TBX10*, *TBR1*). X axis showed the enriched transcription factors and Y axis showed the adjusted *p* value for the motif enrichment. (**D**). Deviation of *EOMES* in the clusters of t(8;21) AML samples analyzed with chromVAR. (**E**). UMAP showed the transcription accessibility of *EOMES*. (**F**). Footprint of subtype-specific transcription factor *EOMES* in T cells_1 and T cells_2. (**G**). Cell communication plot analyzed from scRNA-seq showed the interaction of T cells_1 and T cells_2 with the other cells

Integration of scRNA-seq and scATAC-seq revealed two distinct subsets of T cells, with T cells_1 characterized by the expression of *CCR7* and *LTB*, and T cells_2 marked by *GNLY*, *NKG7* and *GZMB* (Fig. 4A). In our previous longitudinal study of t(8;21) AML [16, 17], we discovered a subset of T cells expressing *GZMB*, *NKG7*, and *GNLY* that expanded during the drug-resistant stage, of which the extracted gene signature was shown to be a potential biomarker of prognosis for AML [17].

To examine the clonality and rearrangements of these two subsets of T cells (T cells_1 and T cells_2), as well as their contributions to leukemia progression, we performed paired scTCR profiling on 1,099 T cells (Fig. 4). We observed significant heterogeneity in the TCR rearrangements and immune repertoire, presenting preferential usage of TCRα chains and TCRβ chains, as well as variable VJ gene combinations, across different AML samples (Fig. S3E).

Next, we assessed the clonality of the T cell populations with reference to previous studies [18] and classified T cells clones according to the frequency into hyperexpanded, large, medium, small and single clones. There was no hyperexpanded or large clones within the two T cells subsets. Furthermore, we observed that T cells_2 exhibited a slightly greater proportions of small and medium clone types compared to T cells_1 (Fig. 4B and Fig. S3F). These results indicated that T cells in t(8;21) AML demonstrated low clonal expansion, with most T cells expressing unique TCRs. Additionally, when calculating the overlap of TCR repertoires to assess their similarity, the Morisita index also indicated minimal overlap, supporting the notion of a diverse and individualized T cell repertoire across these primary AML samples (Fig. S3G).

We next investigated the chromatin regulatory programs underlying the distinct T cell subsets (Fig. 4C). For the T cells_1 cluster, scATAC-seq data showed that *IRF8*, *IRF9*, *ETS1* and *ELK4* were among the predominant transcription factors. *ETS1* has been demonstrated to play a crucial role in CD8 T cell differentiation [19]. In contrast, T cells_2 cluster presented a higher enrichment of transcription factor motifs associated with *EOMES*, *TBR1* and *TBX10*. *EOMES* is responsible for the transcriptional regulation of the exhausted markers *PD-1* and *TIM-3* [20], which can lead to an inferior outcome in AML. The analyses of scATAC-seq data from primary t(8;21) AML further confirmed significantly increased accessibility of *EOMES* within T cells_2 (Fig. 4D, E and F). In addition, T cells_2 presented a weaker cellular communication with other leukemic cells (Fig. 4G). In brief, we identified that T cells_2, which had an epigenetic feature of enrichment of *EOMES* motifs in opening peaks, presented heightened clonality and a propensity for expansion after drug resistance, which might contribute to a poorer prognosis for patients with AML.

### Accessibility of transcription factors that inhibit the differentiation state in primary t(8;21) AML

**Fig. 5:**
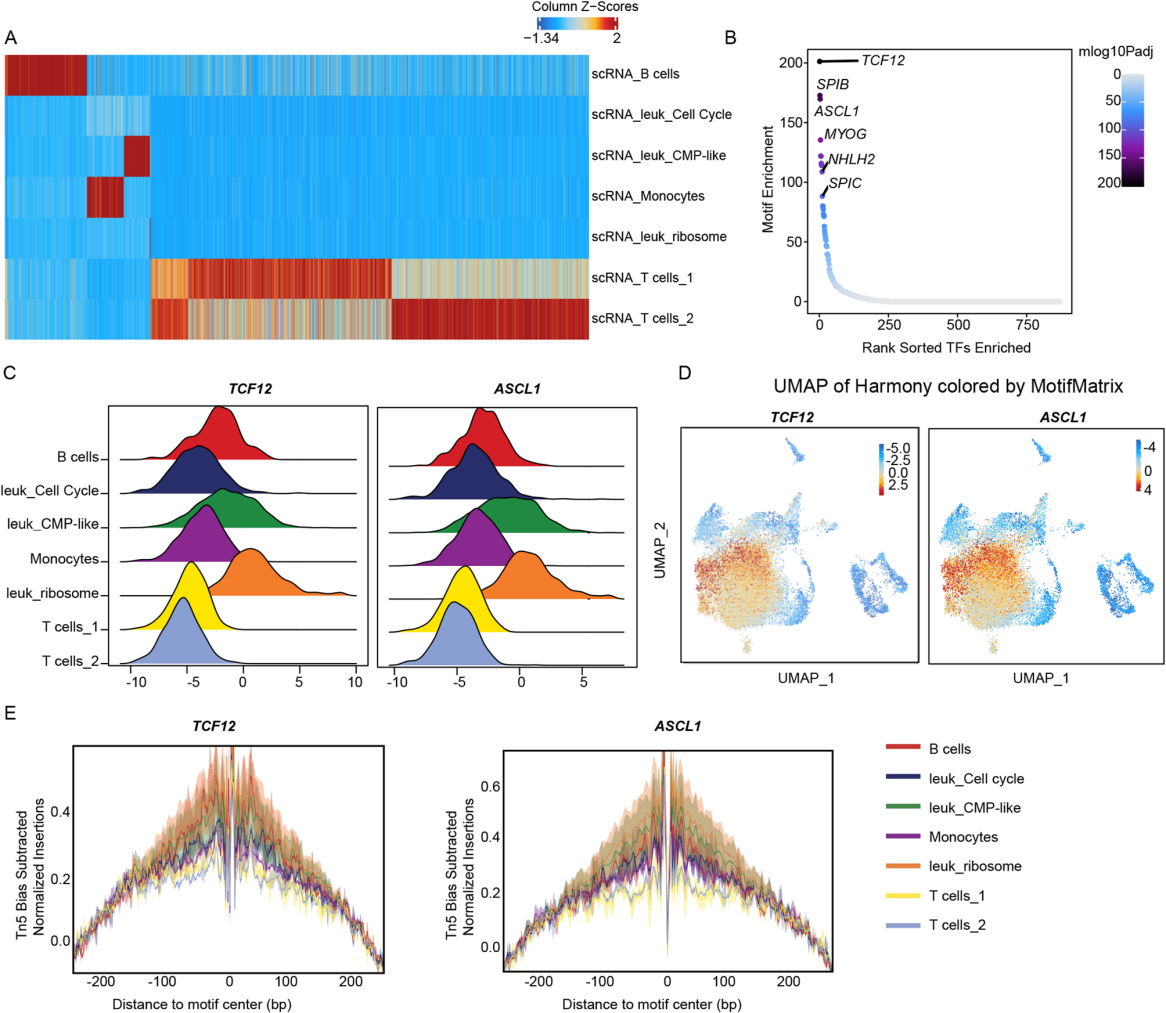
Single-cell chromatin profiling of ribosome leukemia clusters in primary t(8;21) AML. (**a**). Heatmap of peak marker from scATAC-seq showed the inhibited state in leuk_ribosome. (**B**). Plot displayed the top rank transcription factors for ribosome leukemia cells. Among the top including *TCF12*, *SPIB* and *ASCL1*. (**C**). Deviation of *TCF12* and *ASCL1* in the clusters of t(8;21) AML samples analyzed with chromVAR. (**D**). UMAP plot showed the transcription accessibility of *TCF12* and *ASCL1*. (**E**). Footprint of subtype-specific transcription factor *TCF12* and *ASCL1* in the clusters of t(8;21) AML samples

We next explored the epigenetic features across the leukemic cell clusters. Our analysis identified a significant decrease in the overall accessibility of transcription factors within the leukemic cell cluster, indicating that the overall cell differentiation was in an inhibited state in the primitive cell subpopulations (Fig. 5A). scATAC-seq analysis showed that *TCF12* and *ASCL1* were among the top transcription factors within the primitive cell subpopulations (Fig. 5B). Plot tracks and motif matrix analyses demonstrated high accessibility of *TCF12* and *ASCL1* in leuk-ribosome cells (Fig. 5C and D). *TCF12*, also known as *HEB*, participates in the pathogenesis of t(8;21) AML as part of the AETFC complex, and plays an important role in the development of leukemia [3]. Comparative genomic analysis revealed striking similarities in the binding patterns of AML1-ETO and HEB, with both factors exhibiting a preference for promoter-proximal regions and demonstrating comparable distributions relative to transcription start sites (TSS) (Fig. S4A and B). Consistent with these findings, we identified the co-binding of AML1-ETO and HEB at key target loci (including *EP300*, *HDAC7*, *RUNX3*, and *TP53*) (Fig. S4C), providing evidence for their cooperative role in transcriptional regulation. The increased accessibility of TCF12 and its synergistic interaction with AML1-ETO might collectively contribute to the altered regulatory landscape and consequent cellular state alterations observed in t(8;21) AML. In addition, we observed that another transcription factor, *ASCL1*, showed increased accessibility (Fig. 5E), which has not been previously reported. Our research indicated the potential value of *ASCL1* in the pathogenesis in t(8;21) AML.

For the leuk_Cell Cycle cluster, we observed an enrichment of the transcription factors *SP6*, *KLF4* and *WT1* (Fig. S5C-E, Fig. 3C), all of which have been reported to be involved in cell proliferation [21, 22]. The increased transcriptional activity of WT1 facilitated leukemia blasts’ progression into the cell cycle, promoting the transition from G1 to S phase, and ultimately resulting in increased cell proliferation.

### Accessibility of CMP-like leukemia cluster in primary t(8;21) AML

**Fig. 6:**
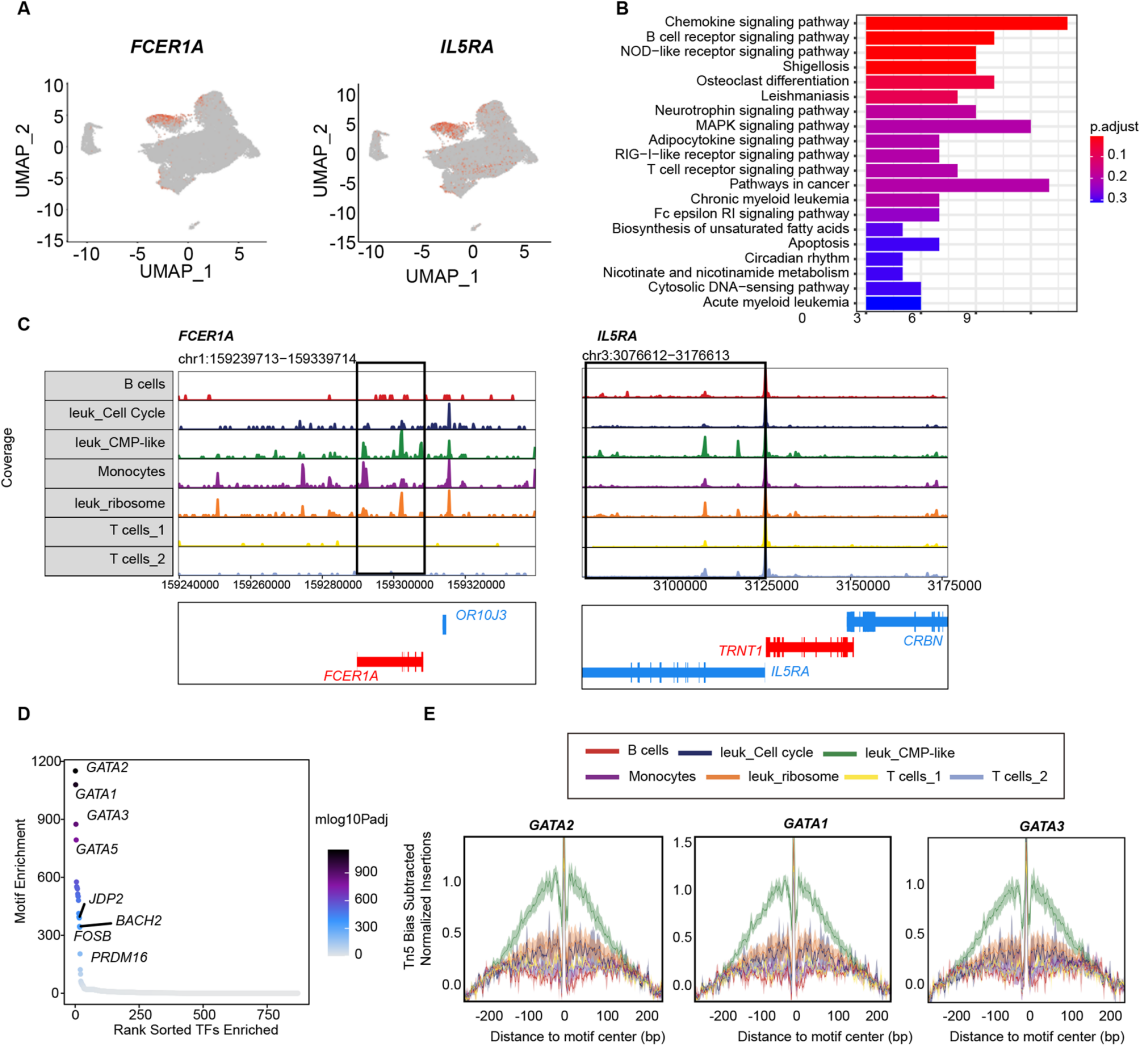
Single-cell chromatin profiling of Leuk_CMP-like cluster in primary t(8;21) AML. (**A**). UMAP plot showed the expression of *FCER1A* and *IL5RA* from scRNA-seq of primary t(8;21) AML (*n* = 3). (**B**). Kyoto Encyclopedia of Genes and Genomes (KEGG) analysis of the top enriched pathways in the Leuk_CMP-like cluster from scRNA-seq. (**C**). Genome browser tracks showed chromatin accessibility of Leuk_CMP-like cluster-specific genes *FCER1A* and *IL5RA* for the cell clusters in scATAC-seq. Each track showed merged signal. (**D**). Plot displayed the top rank transcription factors for Leuk_CMP-like cluster. Among the top including *GATA2*, *GATA1* and *GATA3*. (**E**). Footprint of subtype-specific transcription factor *GATA2*, *GATA1* and *GATA3* in the clusters of t(8;21) AML samples

We identified a distinct cluster of cells, characterized by high expression of *TPSAB1*, *HPGD* and *FCER1A*, designated the leuk_CMP-like cluster. KEGG pathway enrichment analysis revealed the activation of several pathways in this cell population, including the MAPK, chemokine, B cell receptor, and NOD-like receptor signaling pathways (Fig. 6). Joint analysis of scATAC-seq and scRNA-seq data revealed that the epigenetic features of the GATA family members *GATA2*, *GATA1*, *GATA3* and *GATA5* were significantly enriched within the leuk_CMP-like cluster (Fig. 6). These transcription factors are known to play important roles in lineage specification and transdifferentiation in hematologic disorders. We thus hypothesized that the genetic signatures presented within this cluster, whose expression levels are rigorously transcriptionally regulated by the GATA family, might serve as valuable prognostic indicators in AML. To this end, we calculated and extracted the genetic signature of the leuk_CMP-like cluster through joint analyses of scATAC-seq and scRNA-seq data. A distinct gene expression profile of this cluster was defined by genes exhibiting an average log2 (fold change) more than 3.0 and a *p* value less than 0.05. A total of 136 genes were extracted (Table S2), exhibiting stemness- and progenitor-specific characteristics, marked by the high expression of relevant genes associated with AML, such as *CD34*, *CD38* and *RUNX1T1*.

**Fig. 7:**
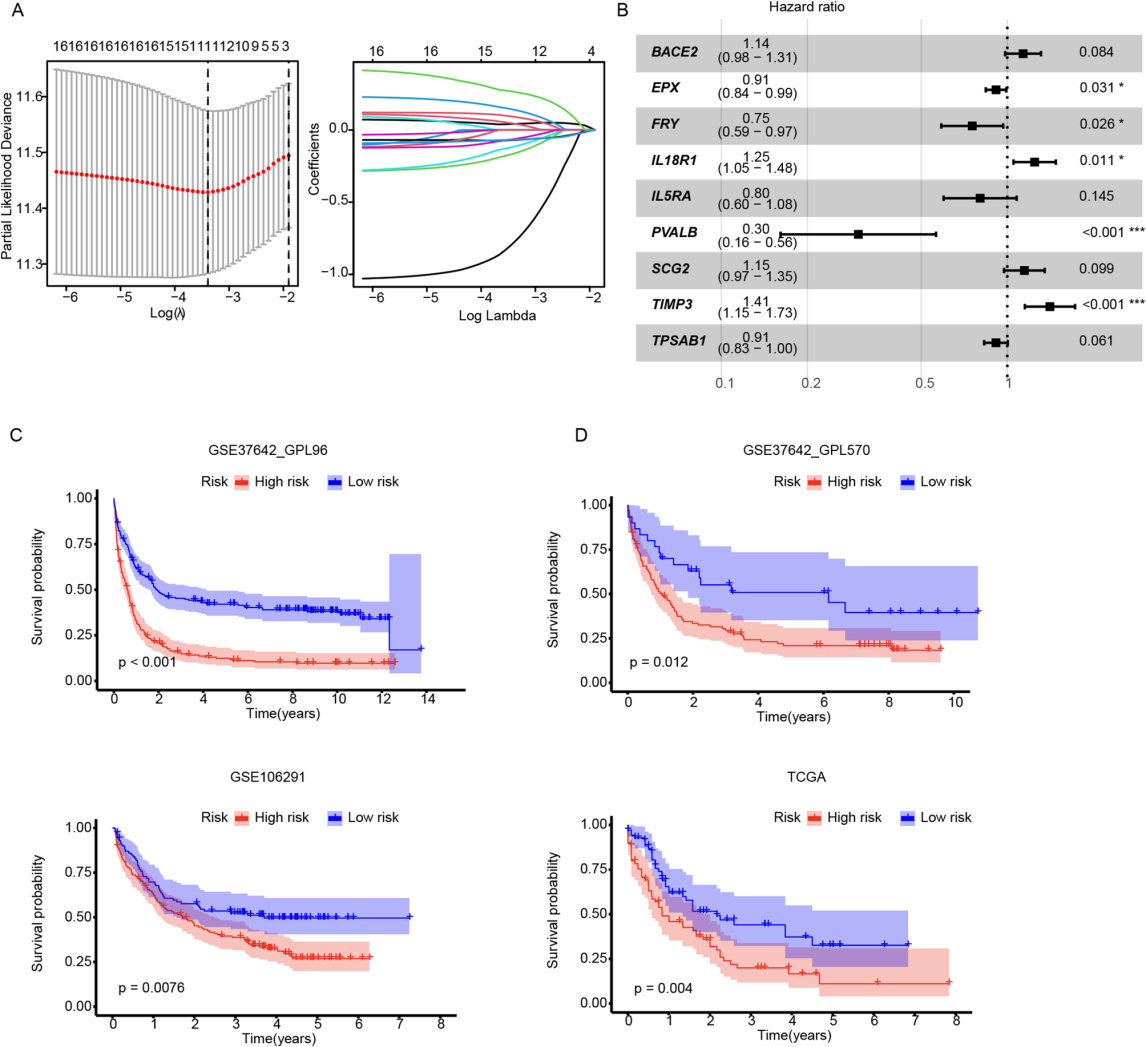
Construction and validation of Leuk_CMP-like cluster related genetic signatures in AML. (**A**). Cross-validation and coefficients of LASSO regression to determine the prognostic model. (**B**). Forest plot showed the Cox regression model. Survival plots of Leuk_CMP-like cluster in training cohort (**C**) and validation cohorts (**D**). Log-rank tests were applied to compare the survival difference in the two risk groups

Lastly, we developed a deconvolution-based prediction procedure to evaluate the potential prognostic value of the leuk_CMP-like signature by utilizing bulk RNA-seq data from the AML cohort. The GSE37642_GPL96 (German AMLCG1999) dataset was used as the training dataset. By employing LASSO regression analysis, we constructed a leuk_CMP-like score derived from the estimated proportions of CMP-like cells in each patient. The final model incorporated nine key genes (Fig. 7A and B), specifically *BACE2*, *EPX*, *FRY*, *IL18R1*, *IL5RA*, *PVALB*, *SCG2*, *TIMP3* and *TPSAB1* (Fig. 7B), of which *TIMP3* and *PVSLB* emerged as strong prognostic indicators of clinical outcome in patients with AML. We subsequently examined the relationship between the Leuk_CMP-like score and the clinical outcomes of AML patients. Remarkably, a high CMP-like score (high-risk subgroup) was significantly associated with inferior overall survival (*P* < 0.001, Fig. 7C) in the training cohort. Moreover, we observed similar findings in the validation cohorts of GSE37642_GPL570, GSE106291 and TCGA LAML (Fig. 7D), reinforcing the prognostic significance of the leuk_CMP-like score.

## Discussion

In this work, we characterized the cellular heterogeneity and global chromatin profiling in AML with t(8;21) translocation. Using scRNA-seq-based gene expression profiling, scATAC-seq-based chromatin accessibility profiling and scTCR-seq-based clonotype clustering, we identified distinct heterogeneous cell populations and explored the epigenetic cis-regulatory mechanism underlying t(8;21) AML. Our study provided the first paired single-cell transcriptomic and epigenomic atlas of t(8;21) AML, addressing critical knowledge gaps in understanding this specific AML subtype. To our knowledge, this study is the first integrative analysis utilizing multiple single-cell technologies to study the cellular and molecular underpinnings of t(8;21) AMLand to compare the results with those of healthy individuals. While previous pan-AML scRNA-seq and scATAC-seq studies have revealed AML cellular heterogeneity and regulatory mechanism, these investigations have either focused primarily on pediatric AML [23], or examined chemotherapy-resistant post-treatment samples [24]. Importantly, none have systematically explored the molecular regulatory mechanisms of the AML1-ETO fusion protein at single-cell resolution in treatment-naïve adult t(8;21) AML patients. Our multi-omics approach uniquely identifies t(8;21) AML-specific regulatory networks and cellular states that were previously obscured in bulk analyses [25].

One of the prominent discoveries of this study was the observation that the family members of the AETFC, namely *TCF12* (*HEB*), *TCF3* (*E2A*) and *LYL1*, presented increased chromatin accessibility in leukemic cells of t(8;21) AML. The AETFC complex plays a crucial role in the regulation of hematopoietic differentiation and the maintenance of multivalent interactions of diverse transcription factors, including the E proteins *HEB* and *E2A*, the AML1-binding partner CBF-β and the hematopoietic E-box-binding transcription factor *LYL1*, all of which contribute to leukemogenesis. Under the pathological state of t(8;21) AML, the AML1-ETO fusion protein aberrantly modulates these transcription factors, leading to increased chromatin accessibility at their target loci. This enhanced chromatin openness likely facilitates the binding of other transcription factors or co-factors, thereby promoting the expression of various target genes that contribute to leukemic transformation. Our findings provide valuable insights into the transcriptional dysregulation underlying the pathogenesis of *AML1*-*ETO*. To therapeutically disrupt the functional cooperation between TCF12/HEB and AML1-ETO, small-molecule inhibitors could be developed to target either their interaction domains or DNA-binding motifs. For example, synthetic peptides mimicking the E-protein interaction interface of AML1-ETO could serve as competitive inhibitors to effectively block this oncogenic partnership [26, 27]. Furthermore, we also identified another transcription factor, *ASCL1*, presenting increased accessibility in leukemic cells in t(8;21) AML patients. *ASCL1* encodes a member of the basic helix-loop-helix (BHLH) family of transcription factors, which acts as a master regulator of neurogenesis and has been shown to be overexpressed in neuroendocrine tumors [28]. It has also been reported as a promising therapeutic target in small cell lung cancer [29]. Although *ASCL1* is a well-established regulator in neuroblastoma and other cancers, its role in hematopoietic malignancies is still under investigation. While ASCL1’s role in AML is emerging, its established function in neuroendocrine cancers suggests the tractability. Pharmacological degradation (e.g., proteolysis-targeting chimeras) or transcriptional suppression (e.g., CRISPR-dCas9) of ASCL1 could be explored, especially given its aberrant accessibility in leukemic cells. Research is ongoing to better understand the molecular mechanisms by which *ASCL1* promotes leukemogenesis in AML [30–33]. The above findings not only highlight the importance of transcriptional regulation in t(8;21) AML, but also provide potential therapeutic targets, as intervening in the activity of these transcription factors or their downstream pathways could offer new strategies for the treatment of t(8;21) AML.

Another critical aspect of AML is the interaction between leukemic cells and the surrounding microenvironment, which plays pivotal roles in disease progression, chemoresistance, and relapse. In our previous work, we reported that a subset of T cells is associated with disease progression in AML [17]. By incorporating scTCR-seq in this study, we further revealed a diverse T cell repertoire in patients with t(8;21) AML at initial onset, with T cell_2 clusters exhibiting an oligoclonal TCR repertoire and presenting key associations with drug resistance. In the T cell_2 cluster, the transcription factor *EOMES* was identified as a pivotal regulatory factor. *EOMES* is known to be involved in the differentiation of cytotoxic T lymphocytes and natural killer cells, playing a critical role in their function and survival [34–36]. Several studies have reported a potential link between *EOMES* and T cell exhaustion, particularly regarding exhaustion markers such as PD-1 and TIM-3 [20, 37, 38]. PD-1 and TIM-3 are key checkpoint molecules that are highly expressed on exhausted T cells, which often accumulate in the tumor microenvironment, thus leading to T cell dysfunction. This observation has important translational implications. Immune checkpoint inhibitors (ICIs) targeting PD-1/PD-L1 or TIM-3 have shown efficacy in solid tumors and hematologic malignancies [39, 40]. Given the association between *EOMES* and these checkpoint molecules, combinatorial strategies, such as *EOMES*-modulating agents alongside ICIs, could be explored to reverse T cell exhaustion and restore anti-leukemic immunity. Additionally, since oligoclonal T cell expansions may indicate antigen-driven responses, identifying the cognate antigens recognized by these T cells could uncover novel targets for immunotherapy, such as TCR-engineered T cells or vaccines.

We also identified a subset of cells exhibiting CMP-like gene characteristics with high expression of marker genes specific to early progenitors, such as *CD34*, *CD38* and *GATA1*, possibly representing leukemic CMP-like cells. Integrated analysis of the scRNA-seq and scATAC-seq data revealed that this cluster highly expressed *TPSAB1*, *HPGD*, and *FCER1A*, which was under the transcriptional regulation of GATA family members, including *GATA2*, *GATA1*, *GATA3* and *GATA5*. A number of studies have reported that the GATA protein family comprises vital pluripotency-associated transcription factors determining the identities of pluripotent and differentiated cells [41–43]. In addition, recent studies demonstrate that *GATA2* dosage controls the generation and maintenance of the hematopoietic stem cell, with overexpression promoting stemness and is more resistant to the standard AML chemotherapy agent doxorubicin [44]. Our results further revealed that the GATA family participated in the epigenetic reprogramming of leukemic cells with chromatin accessibility alterations, and is thus involved in stem cell maintenance and leukemogenesis promotion in t(8;21) AML. The co-expression of *FCER1A* (high-affinity IgE receptor) and *HPGD* (prostaglandin catabolizing enzyme) suggests potential microenvironmental interactions. *HPGD*-mediated regulation might exert tumorigenic effects through non-enzymatic mechanisms [45]. These might create a pro-leukemic niche favoring immune evasion, consistent with our finding of dysfunctional cytotoxic T cells in patients with t(8;21) AML. These findings are consistent with the emerging concept of lineage infidelity in AML [46], wherein epigenetic dysregulation drives cells into aberrant states. Future studies should functionally validate the leukemogenic potential of this CMP-like population through xenotransplantation models and explore whether its prevalence correlates with treatment outcomes.

While our study provides valuable insights into the epigenetic landscape of AML, we acknowledge several limitations that warrant consideration. First, our analysis was restricted to three t(8;21) AML samples and two healthy controls due to the rarity of high-quality, multi-omics-matched specimens. While this sample size allowed us to identify robust cell-type-specific regulatory signatures, larger cohorts would improve statistical power to detect subtle heterogeneity and rare subpopulations. Future studies with expanded sample sizes are needed to validate and generalize our findings across the heterogeneous t(8;21) AML subtypes and clinical contexts.

Addtionally, our scATAC-seq and scRNA-seq analyses were limited to baseline diagnostic samples, which was necessitated by the practical challenges associated with obtaining longitudinal bone marrow specimens. Future investigations incorporating paired diagnosis-relapse samples or serially collected post-treatment specimens would substantially enhance our understanding of how transcriptional and epigenetic reprogramming drives therapeutic resistance and relapse mechanisms, thereby offering opportunities to improve clinical outcomes through more targeted therapeutic strategies. Such investigations could capture the dynamic changes in chromatin accessibility during disease progression or in response to treatment, which is a critical aspect for comprehensively elucidating the epigenetic evolution of AML.

Finally, while single-cell multi-omics technologies offer unprecedented resolution, they are subject to inherent technical limitations, including gene dropout effects in scRNA-seq, sparse coverage in scATAC-seq, and potential batch effects across samples. Computational challenges in integrating multimodal data may also influence interpretability. We mitigated these issues through rigorous quality control, batch correction, and cross-modal integration approaches, but residual confounding factors might persist. Advances in both experimental protocols and bioinformatic pipelines will further enhance the reliability of single-cell studies in t(8;21) AML.

## Conclusions

In summary, this multi-omics study, which integrates scRNA-seq, scATAC-seq and scTCR-seq, establishes a framework for a more comprehensive understanding of the gene expression alterations and transcriptional regulatory networks, as well as the role of immune cell dynamics in TME in t(8;21) AML. The high concordance between transcriptional- and epigenomic-based subtypes, together with the TCR repertoire profiling of the TME, revealed strong biological differences between distinct cell populations. Moreover, the inclusion of a genetic signature could be used as a novel and useful prognostic indicator for patients with t(8;21) AML.

## Electronic supplementary material

Below is the link to the electronic supplementary material.

Supplementary Material 1

## Data Availability

The raw sequencing data reported in this paper have been deposited in the Genome Sequence Archive in National Genomics Data Center (https://ngdc.cncb.ac.cn/gsa-human), under the accession number #HRA007073. Due to the legal restrictions, these data are under controlled access. Requests for access to these data for research purposes can be directed to the corresponding author Dr. Lu Jiang (jl11891@rjh.com.cn).
