# Supplementary figures and images for: A multi-omic single-cell landscape reveals transcription and epigenetic regulatory features of t(8;21) AML
Source: J Transl Med. 2025 Jul 24;23:816. doi: 10.1186/s12967-025-06659-0 (PMC12288317; doi:10.1186/s12967-025-06659-0)

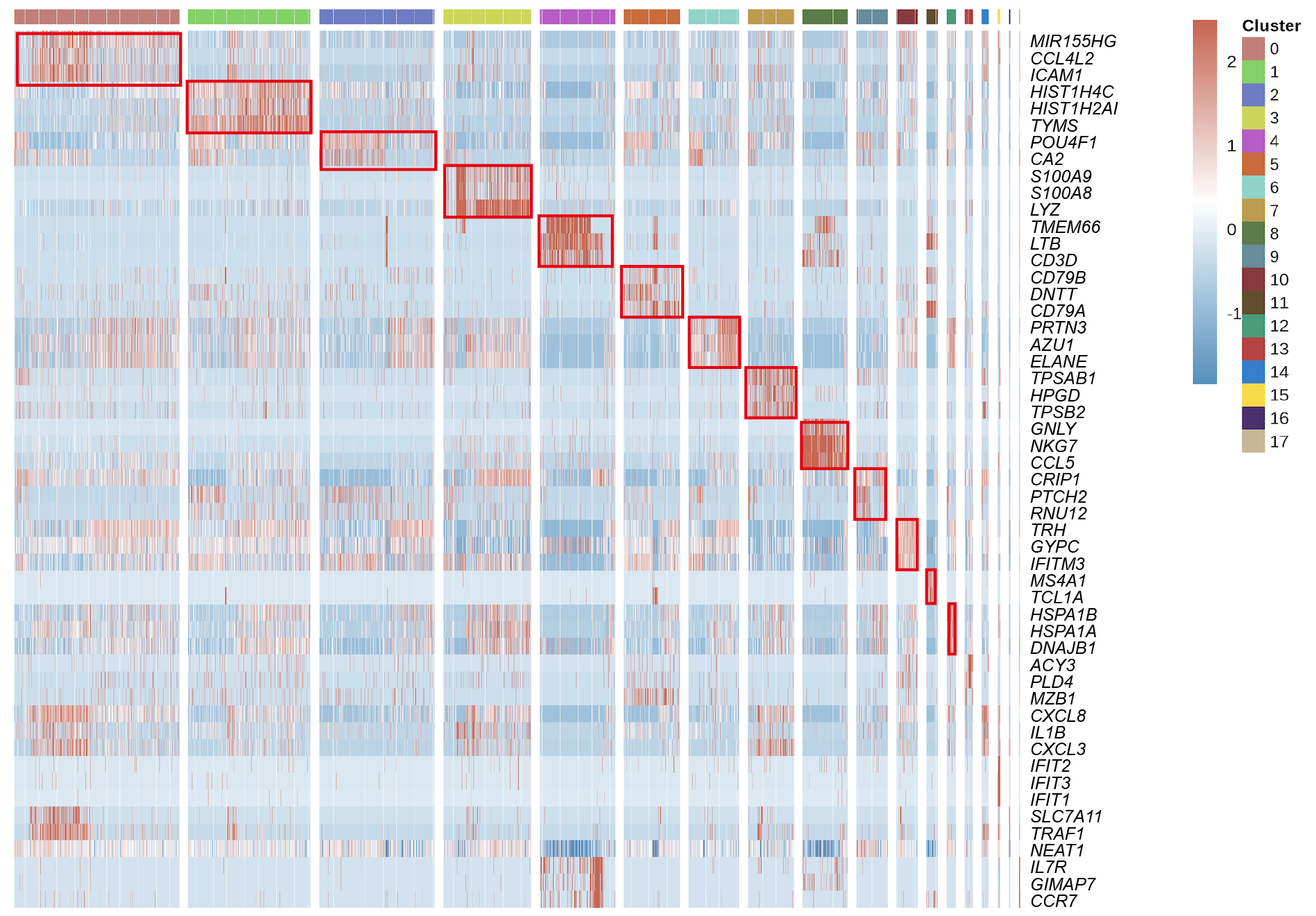

Supplement: Supplementary file 1 — Supplementary Material 1 [file 12967_2025_6659_MOESM1_ESM.zip › 12967_2025_6659_MOESM1_ESM/12967_2025_6659_MOESM1_ESM.tif]

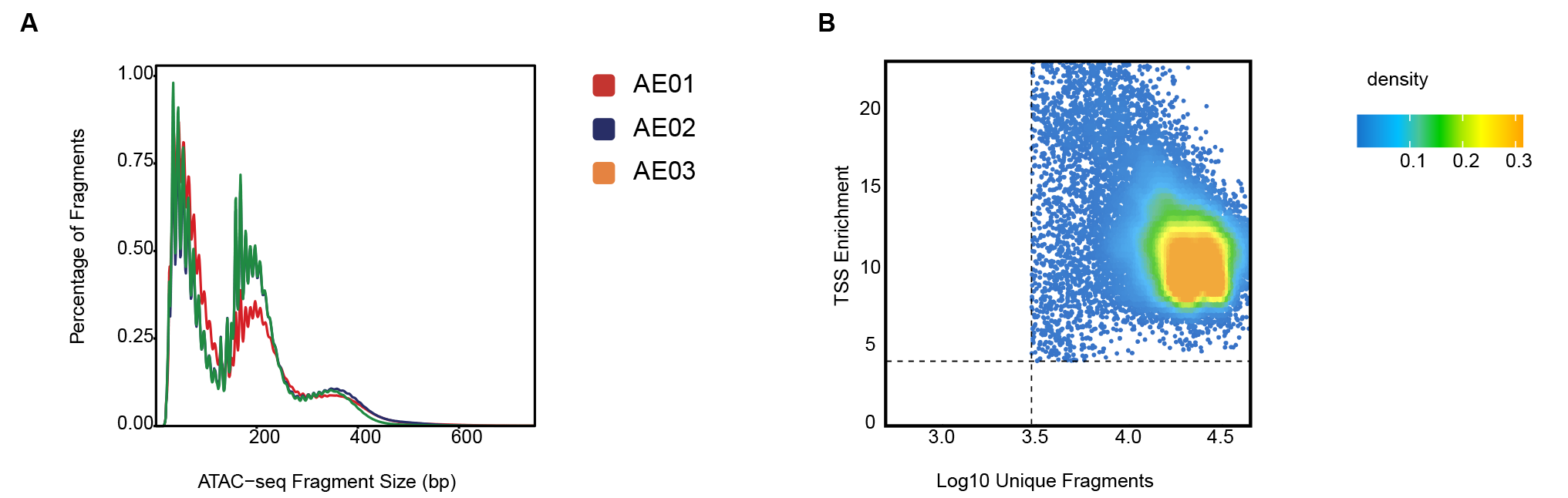

Supplement: Supplementary file 1 — Supplementary Material 1 [file 12967_2025_6659_MOESM1_ESM.zip › 12967_2025_6659_MOESM1_ESM/12967_2025_6659_MOESM2_ESM.tif]

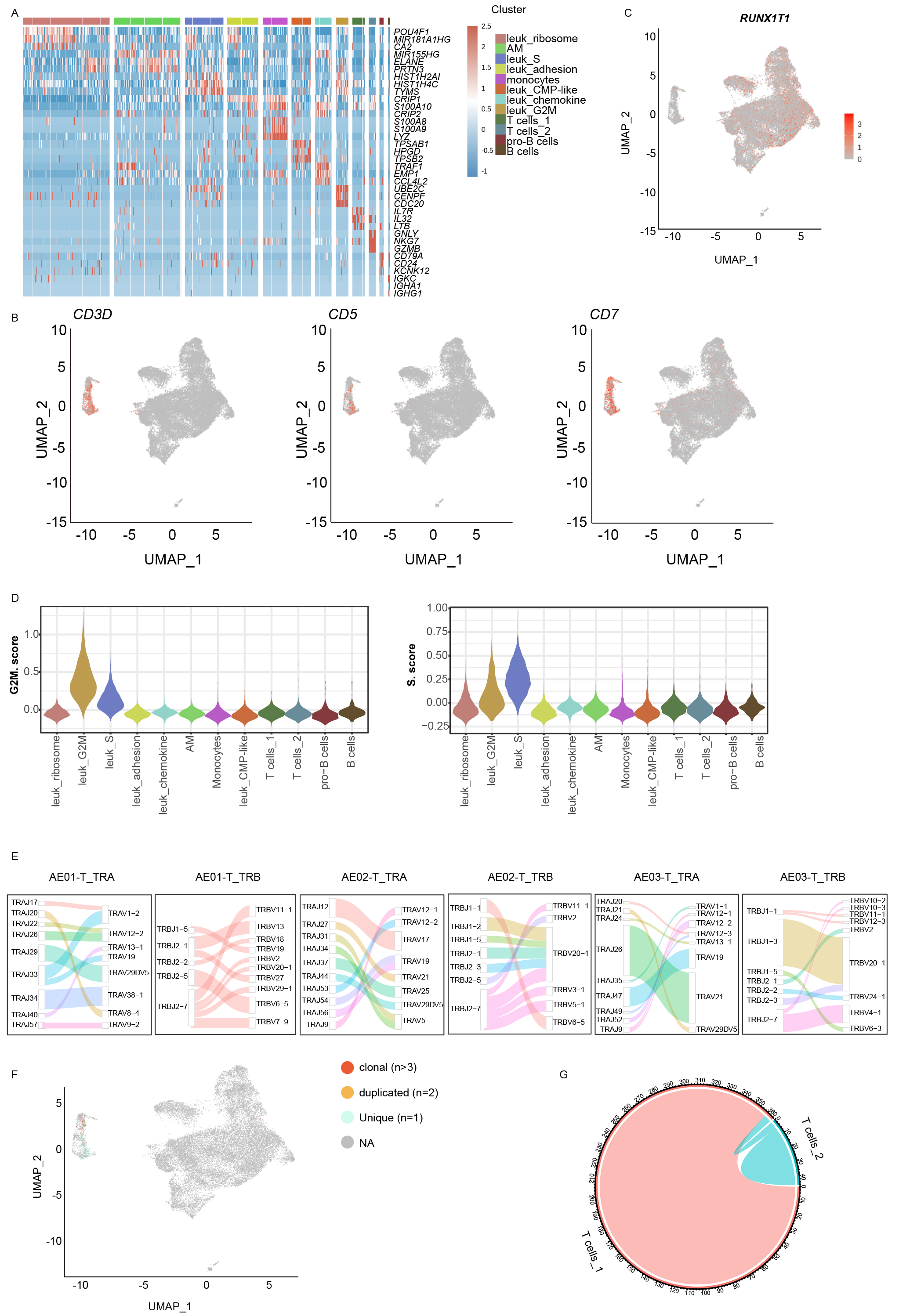

Supplement: Supplementary file 1 — Supplementary Material 1 [file 12967_2025_6659_MOESM1_ESM.zip › 12967_2025_6659_MOESM1_ESM/12967_2025_6659_MOESM3_ESM.tif]

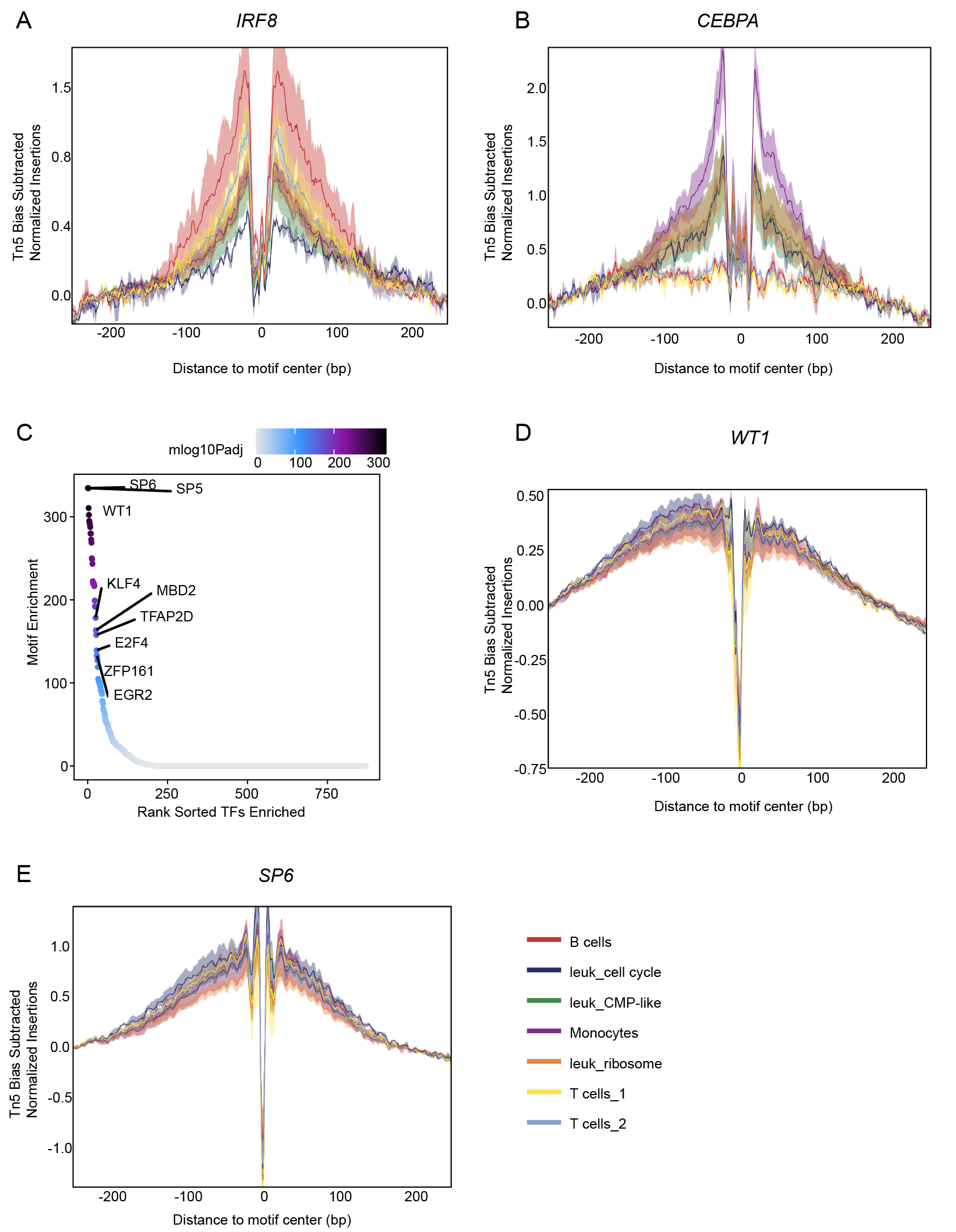

Supplement: Supplementary file 1 — Supplementary Material 1 [file 12967_2025_6659_MOESM1_ESM.zip › 12967_2025_6659_MOESM1_ESM/12967_2025_6659_MOESM4_ESM.tif]

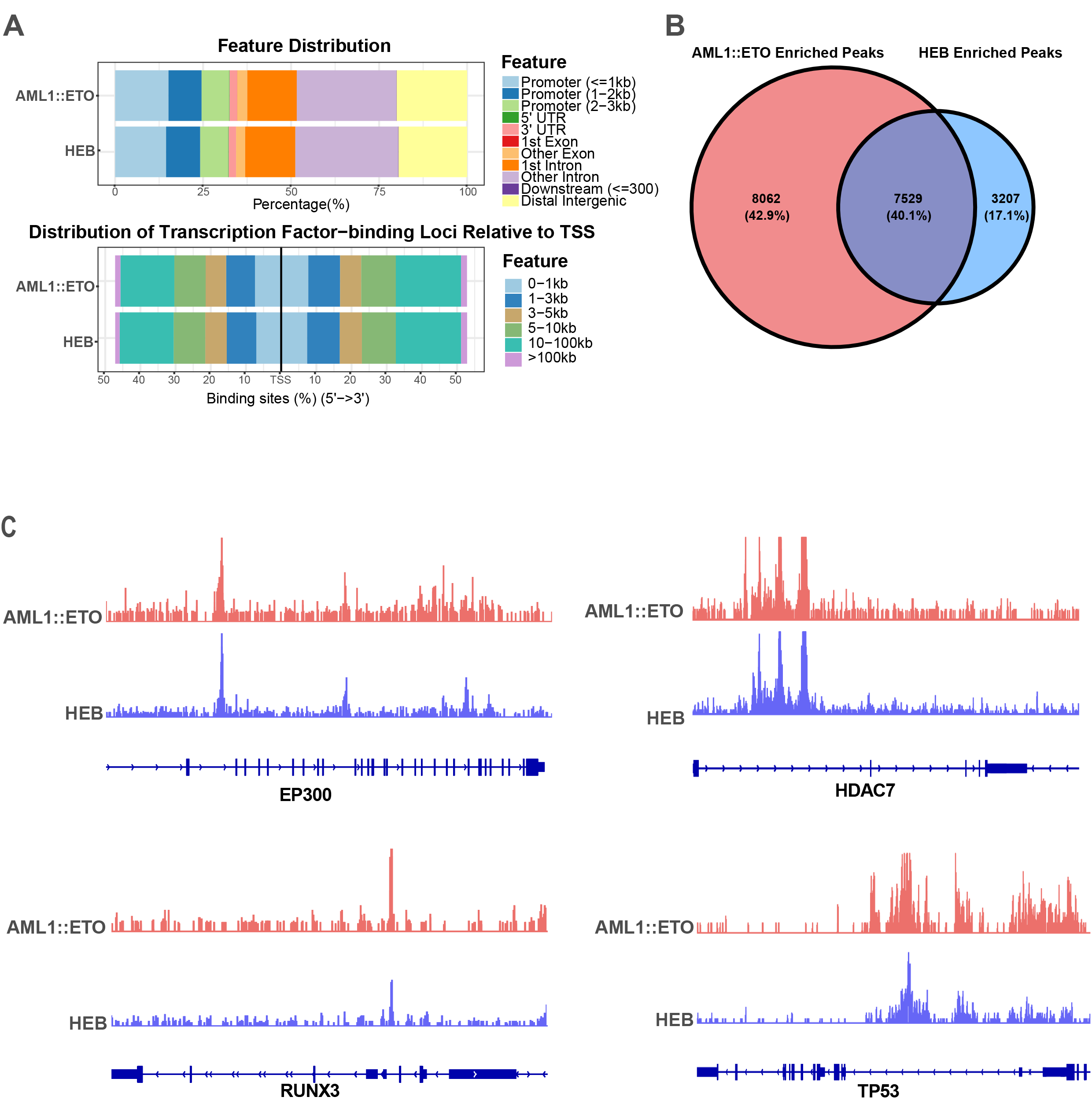

Supplement: Supplementary file 1 — Supplementary Material 1 [file 12967_2025_6659_MOESM1_ESM.zip › 12967_2025_6659_MOESM1_ESM/12967_2025_6659_MOESM6_ESM.tif]
